# PRNP is a pan-cancer prognostic and immunity-related to EMT in colorectal cancer

**DOI:** 10.3389/fcell.2024.1391873

**Published:** 2024-08-05

**Authors:** Haifeng Chen, Yao Du, Zhiyuan Kong, Xinghe Liao, Weiping Li

**Affiliations:** ^1^ Department of Gastroenterology, The First People’s Hospital of Taicang City, Taicang Affiliated Hospital of Soochow University, Taicang, Jiangsu, China; ^2^ Department of Gastrointestinal Surgery, The First Affiliated Hospital of Nanchang University, Nanchang, Jiangxi, China; ^3^ Department of Gastrointestinal Surgery, The First People’s Hospital of Taicang City, Taicang Affiliated Hospital of Soochow University, Taicang, Jiangsu, China; ^4^ Department of Integrated Therapy, Fudan University Shanghai Cancer Center, Shanghai, China; ^5^ Department of Oncology, Shanghai Medical College, Fudan University, Shanghai, China

**Keywords:** PRNP, pan-cancer, prognostic, immunity, EMT

## Abstract

**Background:**

Prion protein gene (PRNP) is widely expressed in a variety of tissues. Although the roles of PRNP in several cancers have been investigated, no pan-cancer analysis has revealed its relationship with tumorigenesis and immunity.

**Methods:**

Comprehensive analyses were conducted on The Cancer Genome Atlas (TCGA) Pan-Cancer dataset from the University of California Santa Cruz (UCSC) database to determine the expression of PRNP and its potential prognostic implications. Immune infiltration and enrichment analysis methods were used to ascertain correlations between PRNP expression levels, tumor immunity, and immunotherapy. Additionally, gene ontology (GO) and Kyoto Encyclopedia of Genes and Genomes (KEGG) methods were employed to examine possible signaling pathways involving PRNP. *In vitro* experiments using CCK-8 assay, Wound healing assay, and Transwell assay to detect the effect of Cellular prion protein (PrPC) on proliferation, migration, and invasion in colorectal cancer (CRC) cells. The expression levels of epithelial-mesenchymal transition (EMT)-related proteins (N-cadherin, E-cadherin, Vimentin and Snail) were detected by western blot.

**Results:**

Among most cancer types, PRNP is expressed at high levels, which is linked to the prognosis of patients. PRNP expression is strongly associated with immune infiltrating cells, immunosuppressive cell infiltration and immune checkpoint molecules. In addition to tumor mutation burden (TMB), substantial correlations are detected between PRNP expression and microsatellite instability (MSI) in several cancers. *In vitro* cell studies inferred that PrPC enhanced the proliferation, migration, invasion, and EMT of CRC cells.

**Conclusion:**

PRNP serves as an immune-related prognostic marker that holds promise for predicting outcomes related to CRC immunotherapy while simultaneously promoting cell proliferation, migration, and invasion activities. Furthermore, it potentially plays a role in governing EMT regulation within CRC.

## 1 Introduction

Cancer is a widespread cause of mortality and significant debilitation, exerting a negative impact on the quality of life globally (Bray et al., 2018). Presently, definitive cures for cancer remain unavailable. Despite considerable progress in treatment modalities, the overall survival (OS) rate for patients after 5 years has remained unsatisfactory despite the use of therapies such as targeted therapy, immunotherapy, and radiation therapy. In 2022, the United States reported new cancer cases of 1,918,030 and 609,360 deaths according to cancer statistics data (Siegel et al., 2022). Conventional strategies have yielded disappointing long-term outcomes despite continuous efforts. However, immune checkpoint blockade therapy has achieved remarkable success as an immunotherapeutic approach to treating cancer (Gao et al., 2020; Herbst et al., 2020). The efficacy of immunotherapy hinges largely upon the identification of specific tumor antigens (Li and Ding, 2020); unfortunately, trials matching immunotherapy-related biomarkers remain limited across most cancers (Khemlina et al., 2017). Fortunately, with the ongoing refinement and development of public databases, like TCGA, the discovery of novel targets for immunotherapy has become more convenient via pan-cancer examination of individual genes, assessing their association with the following aspects; immune infiltration patterns, clinical prognosis, and associated signaling pathways.

Hence, it is crucial to create innovative diagnostic as well as prognostic biomarkers tailored specifically for different types of cancers, given their complexity involving intricate interactions between tumors and the immunological system within the tumor microenvironment (TME). The TME encompasses various cellular components including a substantial proportion of infiltrating immune cells that have a role in initiating and advancing human cancers’ progression (Bindea et al., 2013). The four prion-gene family members constitute PRNP (PrPC), PRND (Doppel), PRNT (PRT), and SPRN (Shadoo). The gene that is widely studied in this family is PRNP(PrPC) (Allais-Bonnet and Pailhoux, 2014). PRNP(PrPC) is predominantly distributed in the central nervous system (CNS), followed by the gastrointestinal tract (Tang et al., 2016; Go and Lee, 2020). Previous studies have confirmed a link between PRNP(PrPC) and the occurrence and development of gastric cancer, CRC, lung cancer, and breast cancer, with its expression being linked to drug resistance, proliferation, apoptosis, migration, and invasion of various malignant tumor cells (Gil et al., 2016; Castle and Gill, 2017; Luo et al., 2017; Atkinson et al., 2019; Lin et al., 2020). The theory is founded on the observation that PRNP(PrPC) can promote anti-apoptosis, invasion, proliferation, and metastasis of cancer cells via different signal transduction pathways and a series of cascading reactions (Li et al., 2010).

We used various publicly available databases to assess the expression of PRNP(PrPC) and the prognostic implications it poses on various cancer types. Next, we investigated potential correlations between PRNP(PrPC) expression levels and both immune infiltrations and the expression of immune checkpoint markers. Additionally, we specifically validated our findings in CRC to examine the associations between PRNP(PrPC) expression levels and the EMT process. The core aim of this research was to determine the potential of PRNP(PrPC) being utilized as a biomarker for predicting the prognosis and the treatment response to immune checkpoint inhibitors, while also elucidating its involvement in CRC’s EMT process. These discoveries provide new insights aimed at improving response rates to immunotherapy.

## 2 Materials and methods

### 2.1 Bioinformatics data and resources

We obtained the TCGA Pan-Cancer dataset, which is a harmonized and standardized collection of data from various cancer types in UCSC (https://xenabrowser.net/). The data on the expression of the PRNP gene (ENSG00000171867) was extracted from this dataset for each sample. Subsequently, the samples were filtered based on their source, and we applied a log2 (x+1) transformation to normalize the expression values. A criterion where the expression level was 0 for the samples was utilized to exclude samples. Furthermore, we performed the same log2 (x+1) transformation and excluded cancer types that were affirmed to have <3 available samples.

### 2.2 Survival analysis

The R packages “survminer” and “survival” were utilized to perform Kaplan-Meier and Cox regression analyses in order to investigate the impact of PRNP on the prognosis of patients. The evaluation focused on OS. A univariate Cox proportional hazards regression model was employed to determine the hazard ratio (HR) for mortalities linked to the expression of PRNP. The adjusted HR for the expression of PRNP was estimated by the constructed multivariate Cox model. *P*-values < 0.05 denoted statistical significance.

### 2.3 Protein-protein interaction (PPI), GO, and KEGG analyses

The PPI network of PRNP was constructed by the STRING database (https://string-db.org/) and enrichment analysis, applying a 0.4 threshold of the minimum interaction score. For the analysis of molecules associated with PRNP, ClusterProfiler packages were used to perform GO and KEGG analyses.

### 2.4 Immune landscape analysis

The level of immune cell infiltration for each tumor was examined depending on gene expression using the “donvo_CIBERSOR” method from the R package IOBR. Infiltration scores of cancer-associated fibroblasts (CAFs), B cells, CD4_T cells, CD8_T cells, Macrophages, NK cells, Endothelial cells, and other cells were reassessed in each tumor using the “donvo_epic” method from the R package IOBR. Furthermore, the B cell infiltration score, T cell CD4 infiltration score, T cell CD8 infiltration score, Neutrophil infiltration score, Macrophage infiltration score, and DC (Dendritic Cell) infiltration score were re-examined for each patient’s tumor as per gene expression using the “Timer” approach that is provided by the R package IOBR. PRNP expression data along with 60 marker genes representing 24 Inhibitory and 36 Stimulatory immune checkpoint pathway genes were analyzed in every sample. The “TMB function” provided by the R package was used to compute the TMB of each tumor. An analysis was then performed to calculate individual tumors’ TMB value by combining TMB and gene expression data from samples through the TMB function that is integrated into the R package. Moreover, a previous study availed MSI scores for each tumor (Bonneville et al., 2017).

### 2.5 Materials and reagents

The National Collection of Authenticated Cell Cultures (Shanghai, China) Supplied the Human CRC cell lines HCT116 and HT29. CCK-8 was purchased from GlpBio (United States), and GEM HCl was obtained from Sigma-Aldrich Chemical Co., Ltd. Materials vital in cell culture, including McCoy’s 5A and fetal bovine serum (FBS) were purchased from Procell Life Science & Technology Co., Ltd. (Wuhan, China) and Serana (Germany). Piperstreptomycin and the Transwell chamber were obtained from Beijing Solarbio Science & Technology Co., Ltd. (Beijing, China) and Merck Millipore (Billerica, Massachusetts, United States), respectively. Cytosine arabinoside (Ara-C) was obtained from Ararat (Canton) Biotechnology Co., Ltd. (Guangzhou, China). PRNP overexpression lentivirus (LV-PRNP), blank control vector lentivirus (LV-control), shRNAs targeting the PRNP gene (shRNA-PRNP), and negative control (shRNA-control) were purchased from Shanghai Nuobai Biotechnology Co., Ltd (NM_000311.5). Supplementary Table S1 shows the sequences of shRNAs. The other reagents used met the analytical grade.

### 2.6 Cell culture

The human CRC cell lines HCT116 and HT29, which exhibit varying levels of PrPC expression, were acquired from the National Collection of Authenticated Cell Cultures and were cultured in McCoy’s 5A medium supplemented with 10% FBS, penicillin (100 U/mL), and streptomycin (100 μg/mL) (P/S). The cells were maintained in the following conditions: a temperature of 37°C and a 5% CO2 humidified atmosphere.

### 2.7 Transfection of recombinant lentivirus with different expression levels of PrPC in tumor cells

HCT116 in conjunction with HT29 tumor cells was cultured after attaining the logarithmic growth phase. Cells were introduced to each well of a 6-well plate at a density of 5 × 10^4^ cells/mL. The wells were then grouped into four: PrPC-, PrPC+, PrPC- control, and PrPC+ control. Following a 12-h culture period, the cells were infected with the lentiviral vector (PrPC- group add shRNA-PRNP lentiviral vector; PrPC+ group add LV-PRNP lentiviral vector; PrPC- control group add shRNA-control lentiviral vector; PrPC+ control group add LV-control lentiviral vector) when they reached approximately 30%–50% confluence. To ensure consistent infection rates, a multiplicity of infection ratios of 100, 10, 10, and 10 were respectively used for each group. Equal amounts of virus particles, empty vector constructs, and culture medium were added to their corresponding wells. After incubating for another 12 h, 0.5 ug/mL concentration of puromycin was added to each group of supernatant. And then after 12 h of cultivation, the supernatant was discarded and replaced with a complete medium. Four days post-transfection, cell counting was performed using bright field microscopy while the count of green fluorescent protein-positive cells was carried out under fluorescent microscopy. Transfection efficiency was calculated based on these counts. Protein detection analysis was conducted once the transfection efficiency exceeded >80% (Wang S. et al., 2012).

### 2.8 CCK-8 assay for cell proliferation

The human CRC cell lines HCT116 and HT29, with different expression levels of PrPC (5 × 10^5^ cells per well) were seeded onto 96-well plates. They were seeded when they were in their logarithmic growth phase and incubated in McCoy’s 5A containing 10% FBS and P/S at 37°C with 5% CO2 saturation. After being cultured in a medium for 6, 12, and 24 h, these CRC cell lines were incubated with 20 μL CCK8 solution for 4 h at 37°C, and the absorbance value at 490 nm was then recorded.

### 2.9 Wound healing assay

The human CRC cell lines HCT116 and HT29, which exhibited varying levels of PrPC expression, were cultured in six-well plates at a density of 5 × 10^5^ cells per well. After completing 24 h of incubation at 37°C, the cells were subjected to mechanical injury by creating two acellular lanes measuring 1 mm in width using plastic pipette tips and Ara-C (0.2 ug/mL) was added to the medium. Subsequently, any detached cell residue was rinsed from the wells. Changes in the wound area over time were monitored utilizing an inverted microscope, and scratch width was taken at the 24-h mark by ImageJ software as per a previously established protocol (Miyazaki et al., 2015).

### 2.10 Cell migration and invasion assay

The experiments on cell migration assays were conducted following previously described methods (Jia et al., 2017). HCT116 and HT29 cells were cultured for 24 h in a medium that did not have serum. To investigate the migratory behavior of human CRC cell lines HCT116 and HT29 with varying levels of PrPC expression, Transwell cell culture chambers with an 8 mm pore size (BD Bioscience) were employed. In brief, 100 μL of serum-free medium with 80,000 cells and Ara-C (0.2 ug/mL) was introduced to the upper chamber, while 600 μL of culture medium with 20% FBS and Ara-C (0.2 ug/mL) was introduced into the lower chamber. Following a 24-h incubation period, the upper chamber was taken off, and any nonmigrating cells from its surface were gently removed by wiping them off using a cotton swab. The cells that migrated were then fixed utilizing methanol for 20 min, stained with Giemsa for 1 h, and subsequently counted under a microscope. In the invasion experiment, the upper chamber, seeded with 5 ×10^4^ cells and 1% FBS-supplemented medium with Ara-C (0.2 ug/mL), was coated with Matrigel (BD Bioscience); meanwhile, the lower chamber had a 20% FBS-supplemented medium with Ara-C (0.2 ug/mL). After being incubated at 37°C with a CO2 concentration of 5% for 24 h, the Transwell chamber was retrieved, and its contents were discarded before being rinsed with calcium-free PBS solution. Afterward, the cells were treated using methanol and to ensure fixation the treatment was allowed to last for 30 min, followed by staining utilizing a solution containing 0.1% crystal violet for 20 min. Any cells that did not migrate were gently eliminated using a cotton swab and subsequently enumerated under microscopic observation.

### 2.11 Western blot analysis

As previously described, N-cadherin, E-cadherin, Vimentin and Snail proteins in the CRC cells (HCT116 and HT29) were determined using western blot analysis (Subramanian et al., 2012). SDS-PAGE separated equivalent amounts of proteins, followed by transferring them onto a 4 PVDF membrane. A 5% skimmed milk blockaded the proteins, along with incubation of the proteins with the primary antibody (PrPC, 1:2,000; Cat. No: 12555-1-AP, Proteintech, United States and N-cadherin, 1:1,000, Cat. No: AF5239, Affinity Biosciences, United States and E-cadherin, 1:1,000, Cat. No: AF0131, Affinity Biosciences, United States and Vimentin, 1:1,000, Cat. No: AF7013, Affinity Biosciences, United States and Snail, 1:1,000, Cat. No: AF6032, Affinity Biosciences, United States) in PBST for 2 h at room temperature. The membrane was incubated with the secondary antibody (Goat Anti-Rabbit IgG HRP, 1:10,000, Cat. No: SA00001-2, Proteintech, United States) in PBST for 1 h. β-actin (1:10,000, Cat. No: 20536-1-AP, Proteintech, United States) was the internal control. ECL kit from Beyotime was utilized to visualize the proteins.

## 3 Results

### 3.1 Prognostic value and aberrant expression of PRNP in pan-cancer

**FIGURE 1:**
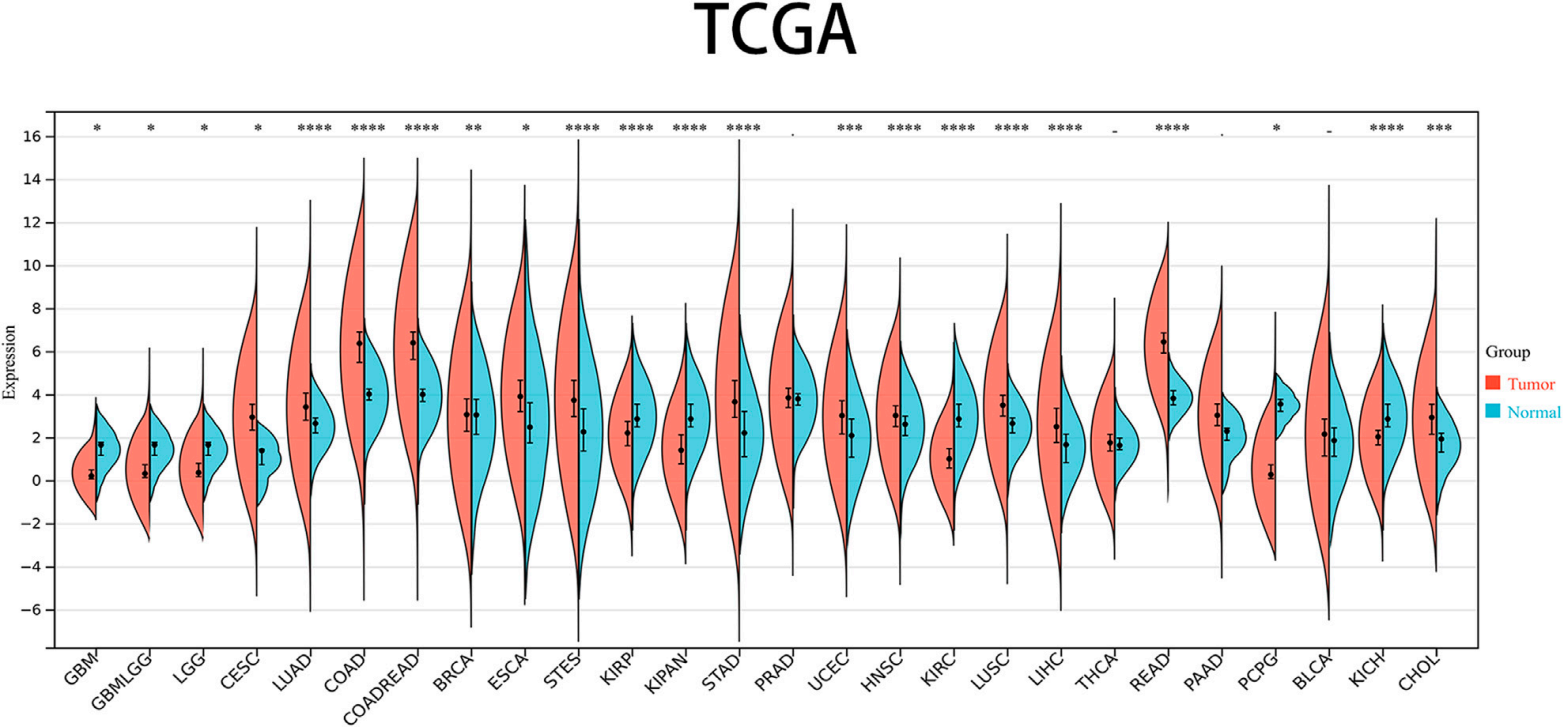
Expression levels of PRNP mRNA in pan-cancer: Comparison of PRNP expression between tumor and normal samples in 26 cancer types obtained from the TCGA database (*p < 0.05; **p < 0.01; ***p < 0.001; ****p < 0.0001; -, not significant).

**FIGURE 2:**
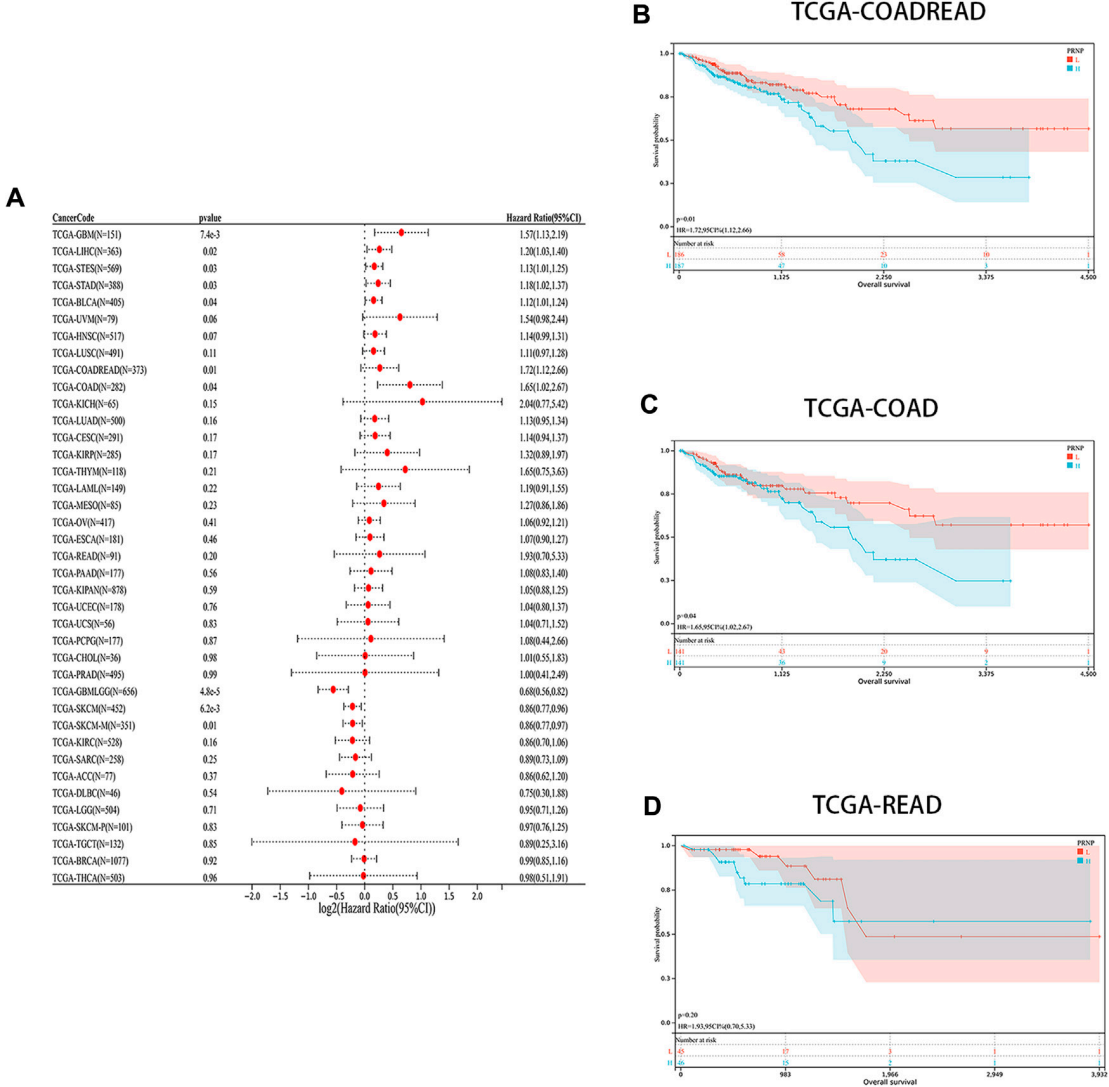
Prognostic analysis of PRNP in different cancer types: **(A)** Forest plot showing the correlation between PRNP expression and OS in various cancers; **(B–D)** Kaplan-Meier survival analysis results show that OS is decreased in COADREAD, COAD, and READ patients with higher PRNP expression levels.

The levels of PRNP mRNA expression in 26 distinct types of cancer were initially examined to assess the significance of PRNP in malignancies. This analysis was conducted using a comprehensive dataset obtained from the TCGA Pan-Cancer (PANCAN) project within the UCSC database. Our findings revealed that PRNP is significantly upregulated in most cancers, with only a few exceptions (Figure 1). To further ascertain the prognostic value of PRNP expression across various cancer types, a survival analysis was conducted employing a Cox proportional hazards model. The results affirmed that heightened levels of PRNP expression were linked to poorer OS rates, specifically in COAD, READ, and COADREAD (Figure 2A). These observations were subsequently confirmed through Kaplan-Meier survival analysis (Figures 2B–D).

### 3.2 Immune infiltration examination of PRNP in pan-cancer

**FIGURE 3:**
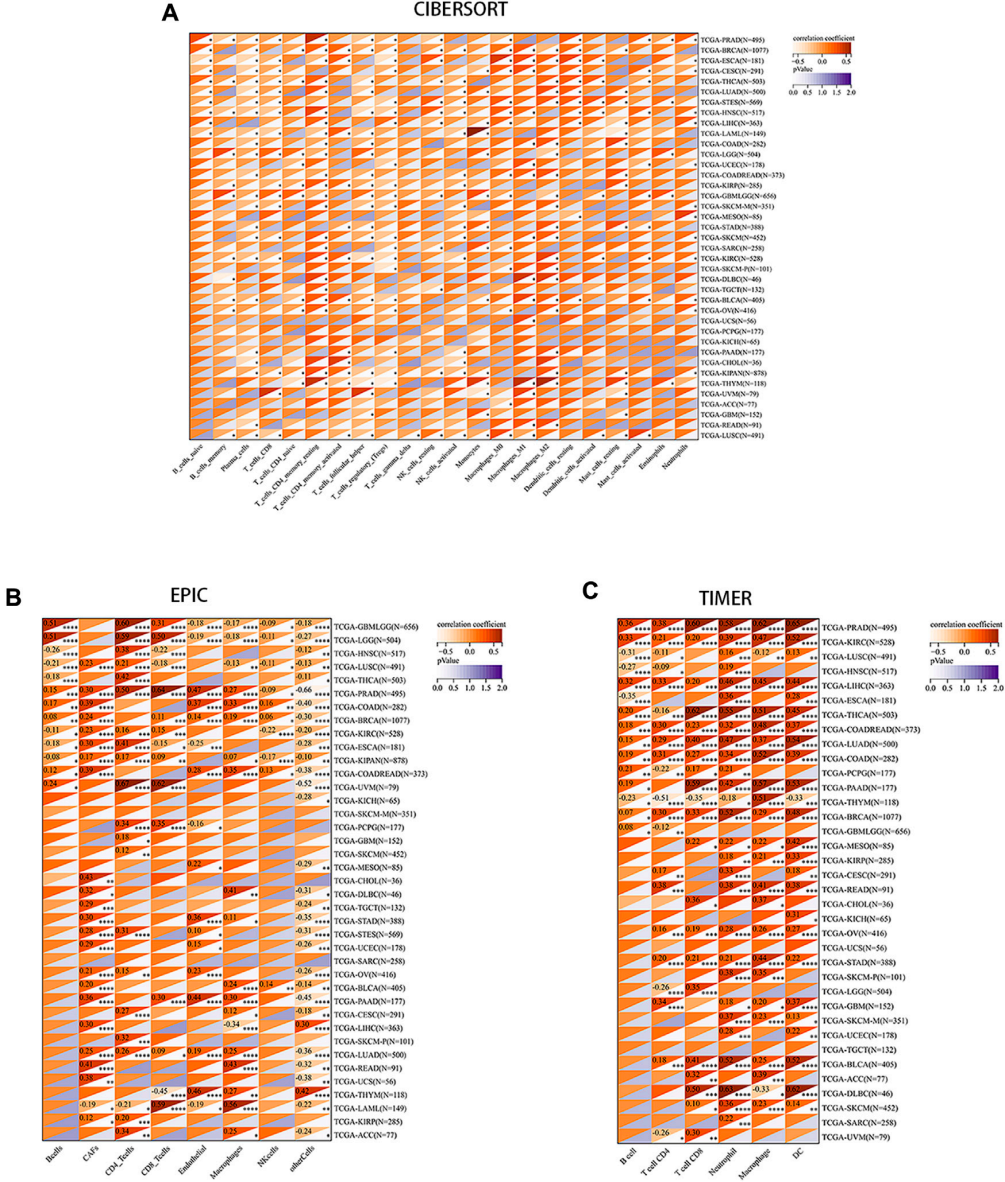
Correlation between PRNP expression levels and immune cell infiltration in various pan-cancer types using: **(A)** The CIBERSORT algorithm; **(B)** The EPIC algorithm; **(C)** The TIMER algorithm.

We aimed to explore the potential of PRNP serving as a therapeutic target for immunotherapy in cancer by investigating its association with tumor immunity. Recognizing the crucial role of TME in tumorigenesis and progression, various algorithms (CIBERSORT, EPIC, and TIMER) were utilized to evaluate the correlation between PRNP expression and immune cell infiltration levels. Findings from these algorithms indicated a positive relationship between PRNP expression and several cell types, including macrophages, neutrophils, CAFs, and CD8^+^ T cells. Conversely, a negative link was observed with plasma cells and regulatory T cells (Figure 3).

**FIGURE 4:**
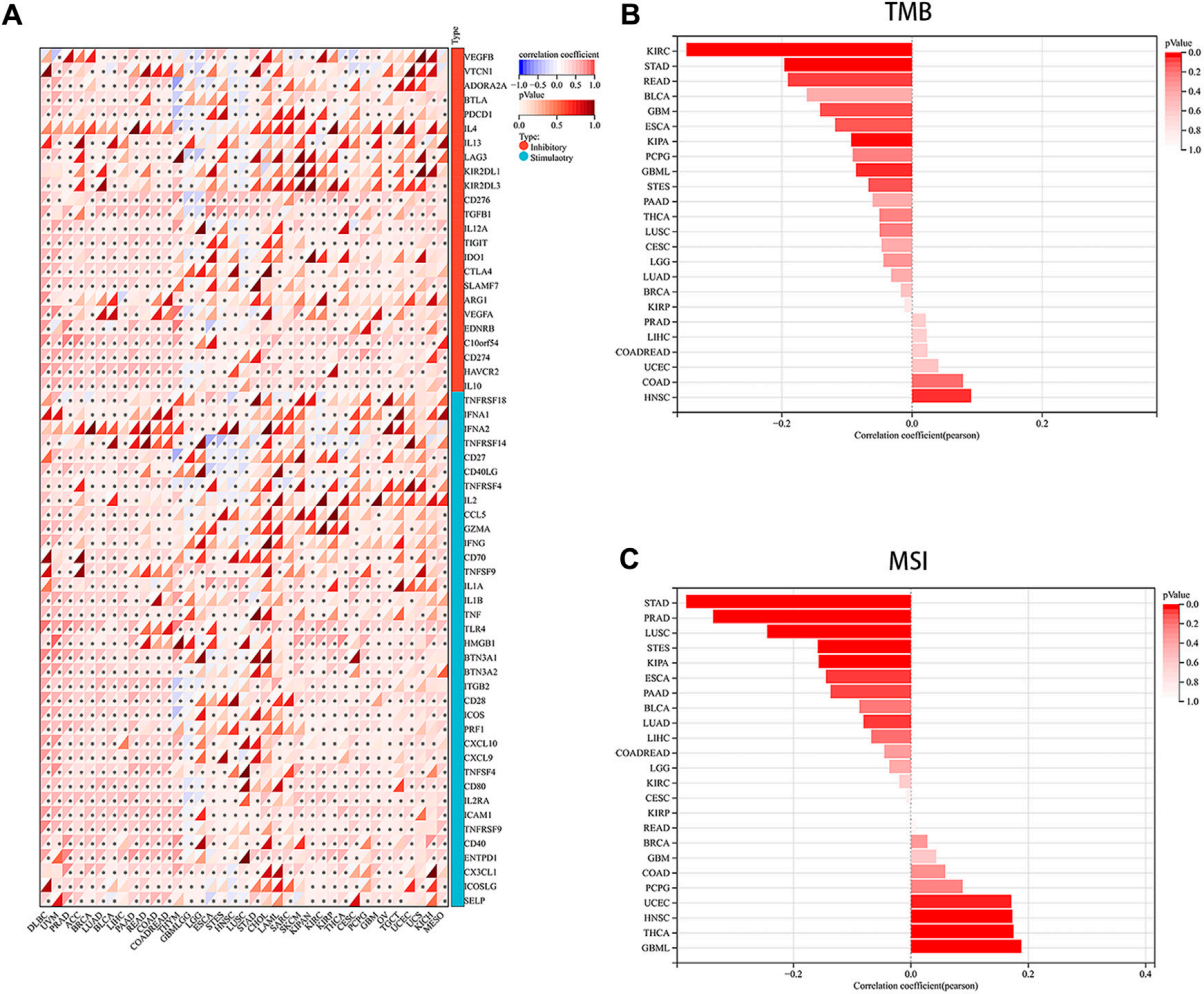
Overexpressed PRNP is positively correlated with immune checkpoint molecules in multiple cancers: **(A)** Heatmap showing correlations between PRNP expression and immune checkpoint genes’ RNA levels in the TCGA pan-cancer database using Spearman’s correlation test; **(B)** Histogram showing the correlation between PRNP expression and TMB in different cancers; **(C)** Histogram showing the correlation between PRNP expression and MSI in different cancers. *p < 0.05.

Immune checkpoints, a crucial mechanism used by tumor cells to avoid detection and attack by T cells, were the focus of our study. We aimed to explore the associations between PRNP and 60 common immune checkpoint expressions (24 inhibitory and 36 stimulatory) across various cancers. Interestingly, positive correlations were found between PRNP and a majority of immune checkpoint molecules in nearly all types of cancer, particularly with CD274 and C10orf54, as shown in Figure 4A. Strong associations were observed between the expression level of PRNP and MSI as well as TMB across distinct cancer types. Specifically, a positive correlation was identified between PRNP and TMB in HNSC and COAD, while in KIRC, STAD, READ, KIPA, and GBML, it showed a negative correlation. Furthermore, a positive link between PRNP and MSI was found in GBML, THCA, HNSC, and UCEC; conversely, a negative correlation with MSI was observed in STAD, PRAD, LUSC, STES, KIPA, ESCA, PAAD, and LUAD, as depicted in Figures 4B, C.

### 3.3 Evaluation of enrichment of PRNP-related genes in pan-cancer

**FIGURE 5:**
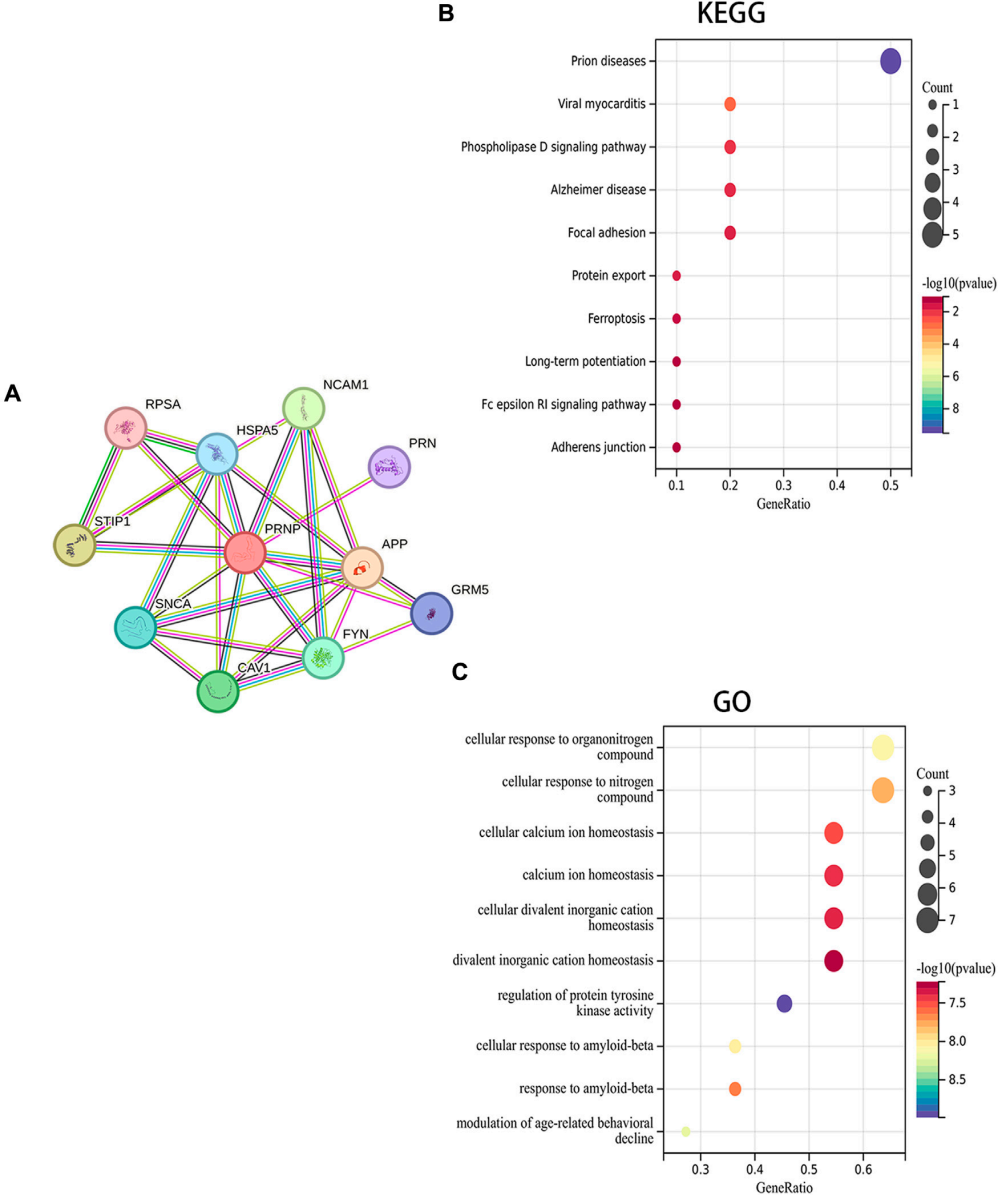
Fuctional enrichment analysis of PRNP-related genes: **(A)** PPI network for PRNP using the STRING datase; **(B)** KEGG and **(C)** GO enrichment analyses of the PRNP-related genes.

A PPI network for PRNP was generated, and the STRING was utilized to detect the top 10 associated genes (Figure 5A). GO and KEGG analyses for the genes followed. The KEGG analysis affirmed a significant enrichment of these genes in pathways such as Prion diseases, Viral myocarditis, and Phospholipase D signaling pathway, among others (Figure 5B). Additionally, the GO analysis demonstrated the enrichment of these genes in cellular responses to organonitrogen compounds, nitrogen compounds, and cellular calcium ion homeostasis, among others (Figure 5C).

### 3.4 Validation of the effect of knockdown and overexpression of PrPC in CRC cell lines

**FIGURE 6:**
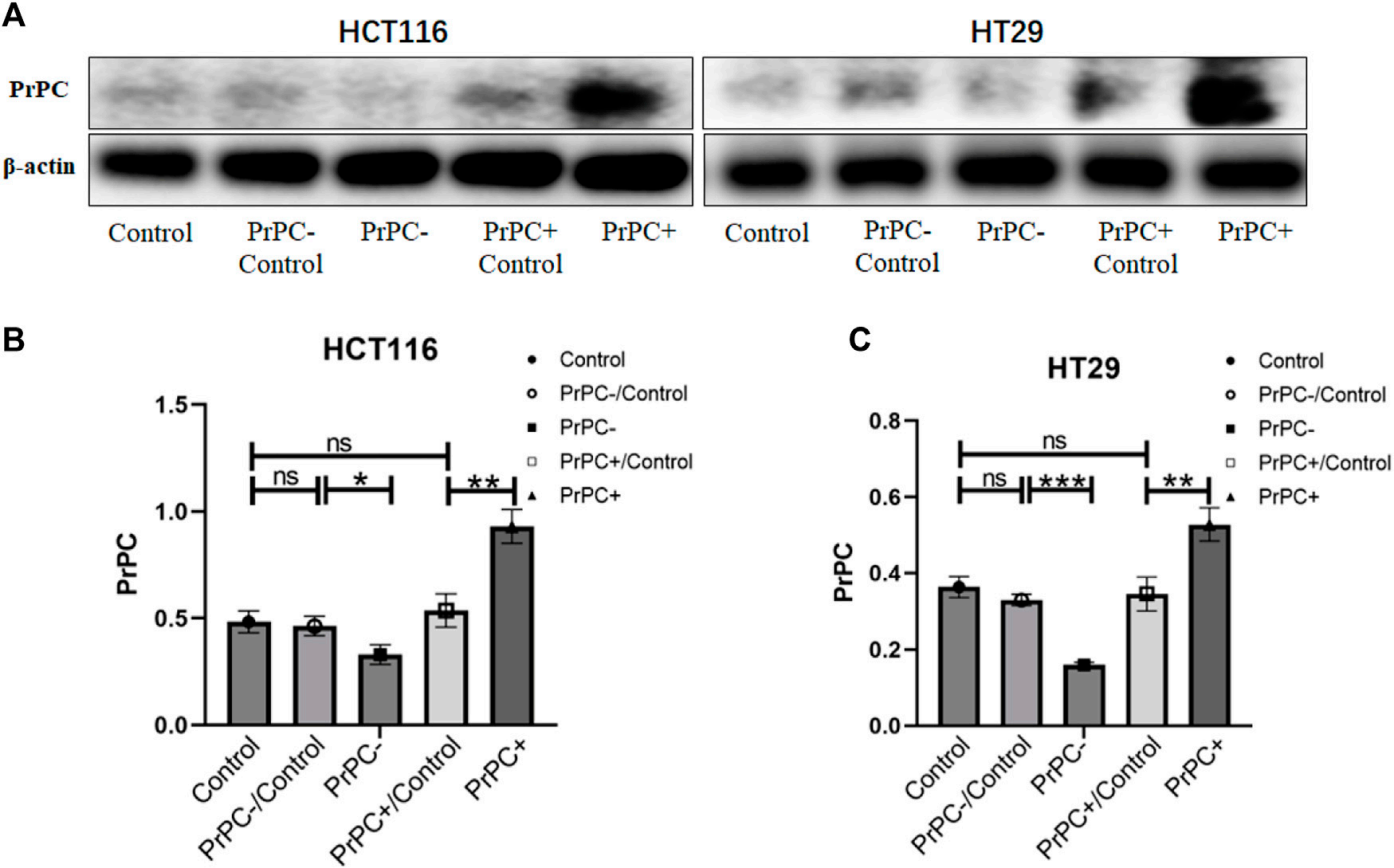
The expression levels of PrPC on CRC cell lines (HCT116 and HT29) and the control group were detected by western blot analysis. ns, no significance. *p < 0.05, **p < 0.01, ***p < 0.001 for comparison with the control group in **(A–C)**.

The transfection efficiency of PrPC- and PrPC+ was analyzed by western blot (Figure 6A). Results showed a statistically substantial decline in the expression of PrPC in HCT116 cells of the PrPC- group compared with the PrPC- control group (*p* < 0.05, Figure 6B). Moreover, a statistically substantial increase in the expression level of PrPC was observed in the PrPC+ group compared to the PrPC+ control group (*p* < 0.01). No significant variation was deduced between the PrPC- control group (or PrPC+ control group) and the blank control group (*p* > 0.05). Similarly, the expression of PrPC in HT29 cells (Figure 6C) decreased remarkably in the PrPC- group (*p* < 0.001) and increased significantly in the PrPC+ group when compared to the corresponding control group (*p* < 0.01).

### 3.5 Effects of different PrPC expression levels on the proliferation ability of CRC cell lines

**FIGURE 7:**
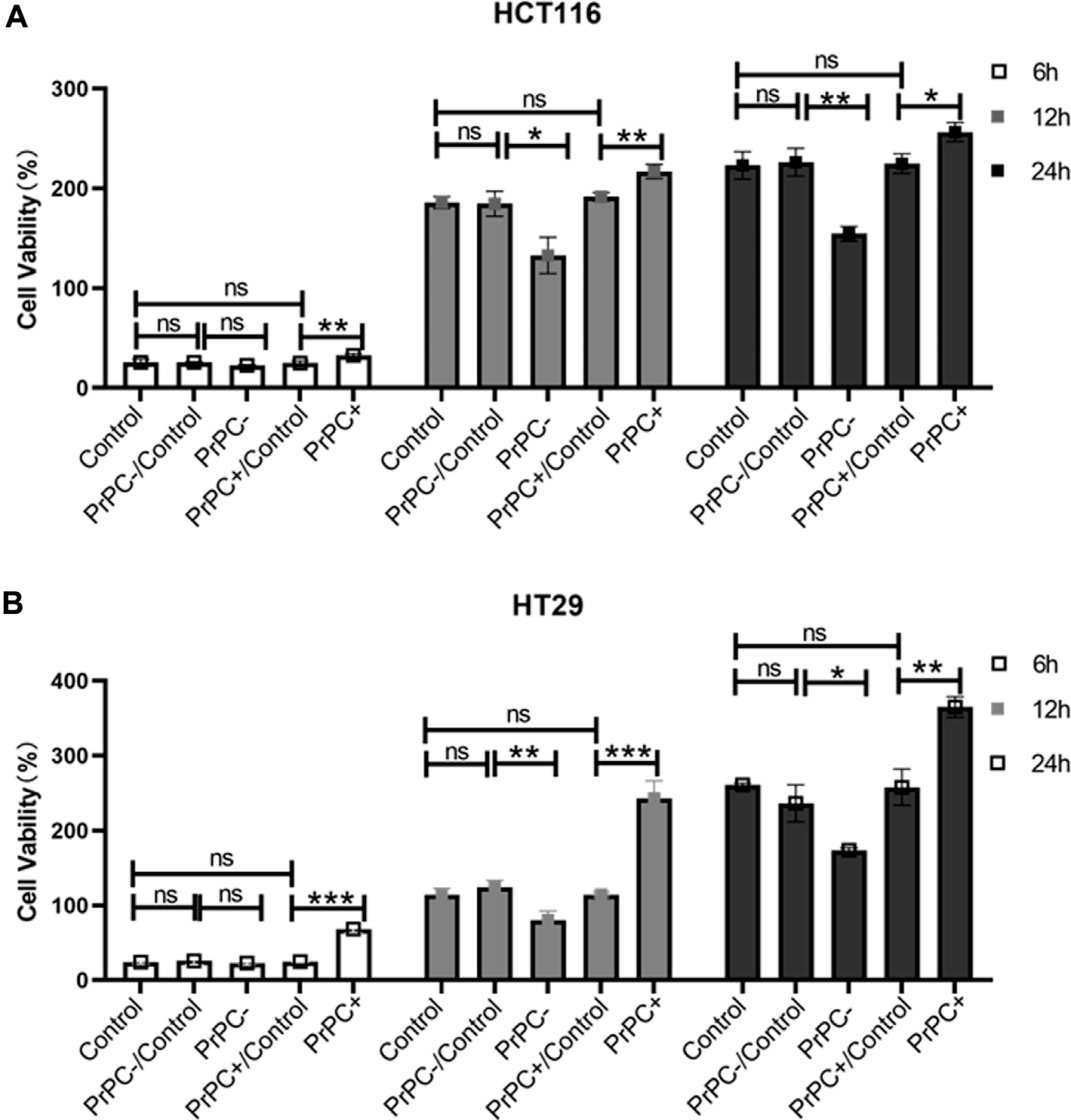
The proliferation of PrPC on CRC cell lines and the control group were detected by the CCK-8 assay. ns, no significance. *p < 0.05, **p < 0.01, ***p < 0.001 for comparison with the control group in **(A, B)**.

HCT116 and HT29 human CRC cell lines were cultured for 6, 12, and 24 h. A CCK-8 assay explored the influence of different PrPC expression levels on CRC proliferation. As shown in Figure 7A, after 6 h of culturing HCT116 cells, no significant difference in cell proliferation was affirmed between the PrPC- control group and the PrPC- group (*p* > 0.05). Nevertheless, after 12 and 24 h of culturing, the proliferation capacity decreased by 28.54% and 30.74% in PrPC- group compared to control group (*p* < 0.05, *p* < 0.01). Conversely, the proliferation ability of HCT116 cell lines significantly increased after 6, 12, and 24 h of culturing when the PrPC gene was overexpressed, and the proliferation capacity increased by 27.05%, 16.72%, and 15.07% in PrPC+ group compared to control group (*p* < 0.01, *p* < 0.01, *p* < 0.05). Similar findings were noted in HT29 cells, after 12 and 24 h of culturing, the proliferation capacity decreased by 29.33% and 33.46% in PrPC- group compared to control group (*p* < 0.01, *p* < 0.05). On the contrary, the proliferation capacity increased by 180.9%, 113.27%, and 39.89% in PrPC+ group compared to control group (*p* < 0.001, *p* < 0.001, *p* < 0.01) (Figure 7B).

### 3.6 Effect of different PrPC expression levels on migration ability of CRC cell lines

**FIGURE 8:**
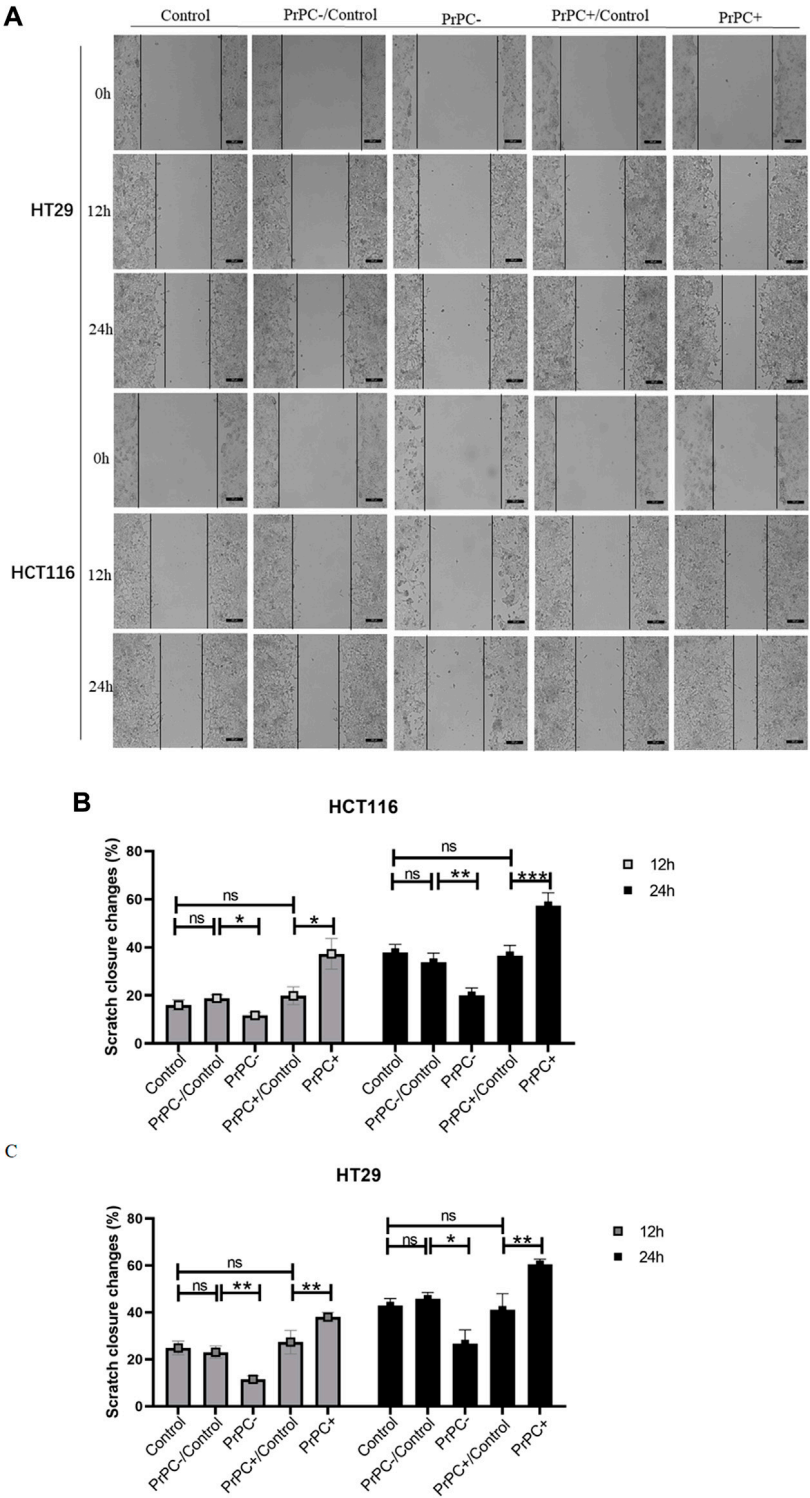
The migration of PrPC on CRC cell lines and the control group were detected by the Wound healing assay. ns, no significance. *p < 0.05, **p < 0.01, ***p < 0.001 for comparison with the control group in **(A–C)**.

**FIGURE 9:**
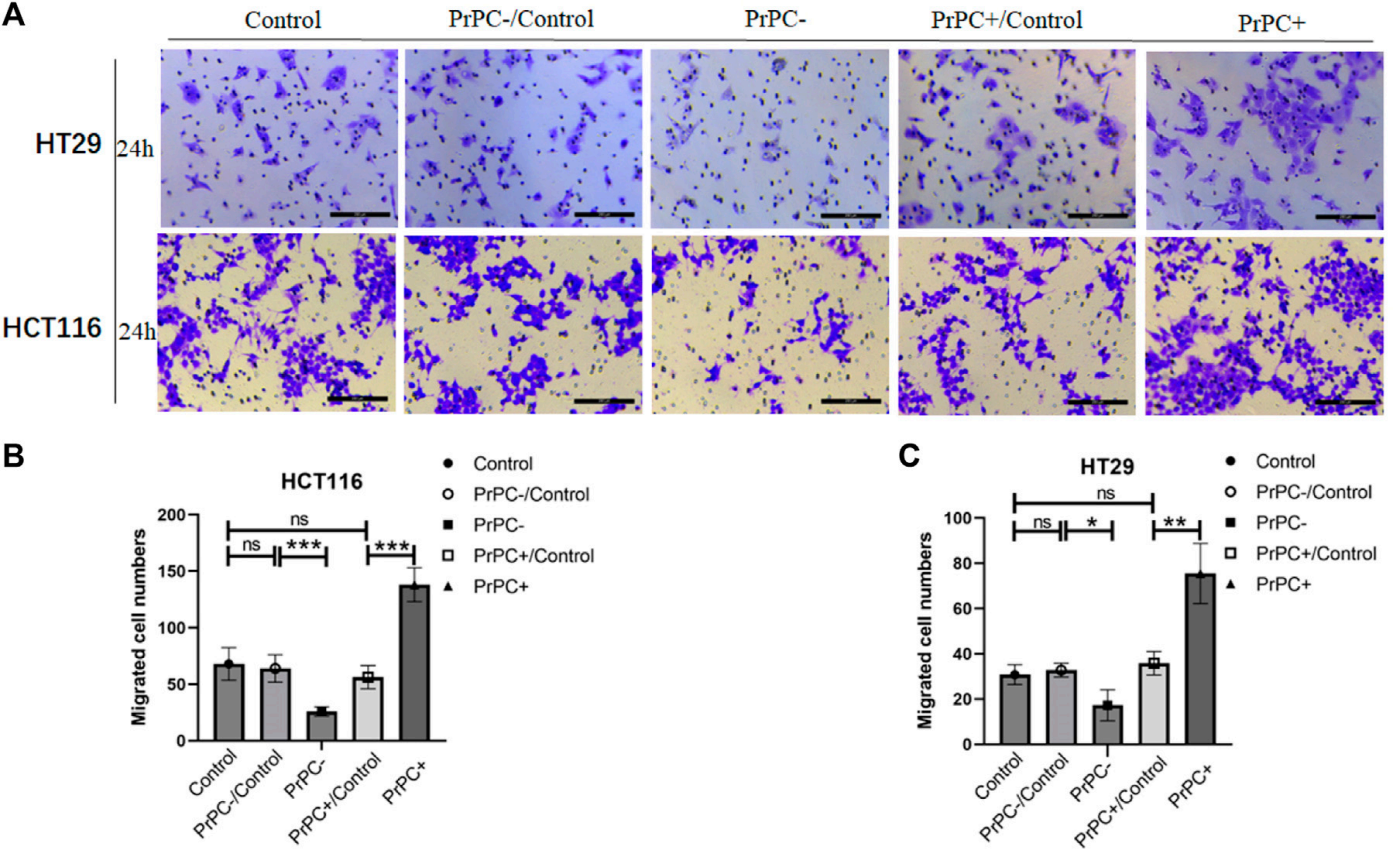
The migration of PrPC on CRC cell lines and the control group were detected by the Transwell assay. ns, no significance. *p < 0.05, **p < 0.01, ***p < 0.001 for comparison with the control group in **(A–C)**.

*In-vitro* cell migration assays were utilized to explore the effect of different PrPC expression levels on CRC migration (Figure 8A). As shown in Figure 8B, results from the Wound healing assay indicated a significant decrease in the migration ability of HCT116 cell lines after 12 and 24 h of cell culture when the PrPC gene was silenced, and the migration ability decreased by 11.72% and 19.99%, respectively (*p* < 0.05, *p* < 0.01). Conversely, the migration ability of HCT116 cell lines significantly increased after 12 and 24 h of cell culture when the PrPC gene was overexpressed, and the migration ability increased by 37.31% and 57.42%, respectively (*p* < 0.05, *p* < 0.001). Similar outcomes were found in HT29 cells, after 12 and 24 h of cell culture, the migration ability decreased by 11.49% and 26.68% in PrPC- group compared to control group (*p* < 0.01, *p* < 0.05). On the contrary, the migration ability increased by 38.06% and 60.40% in PrPC+ group compared to control group (both *p* < 0.01) (Figure 8C). The Transwell cell migration assay was employed to validate the findings of the Wound healing assay regarding the effects of PrPC on CRC (Figure 9A). Data analysis revealed a substantial decline in the number of migrating HCT116 cells in the PrPC- group in comparison to that in the PrPC- control group, and the migration ability decreased by 26% (*p* < 0.001). Conversely, the number of cells migrating in the PrPC+ group was markedly heightened compared to that in the PrPC+ control group, and the migration ability increased by 138% (*p* < 0.001) (Figure 9B). Similar trends were observed in HT29 cells, the migration ability decreased by 17.30% in PrPC- group compared to control group (*p* < 0.05), and the migration ability increased by 75.50% in PrPC+ group compared to control group (*p* < 0.01) (Figure 9C).

### 3.7 Effects of different PrPC expression levels on the invasion ability of CRC cell lines

**FIGURE 10:**
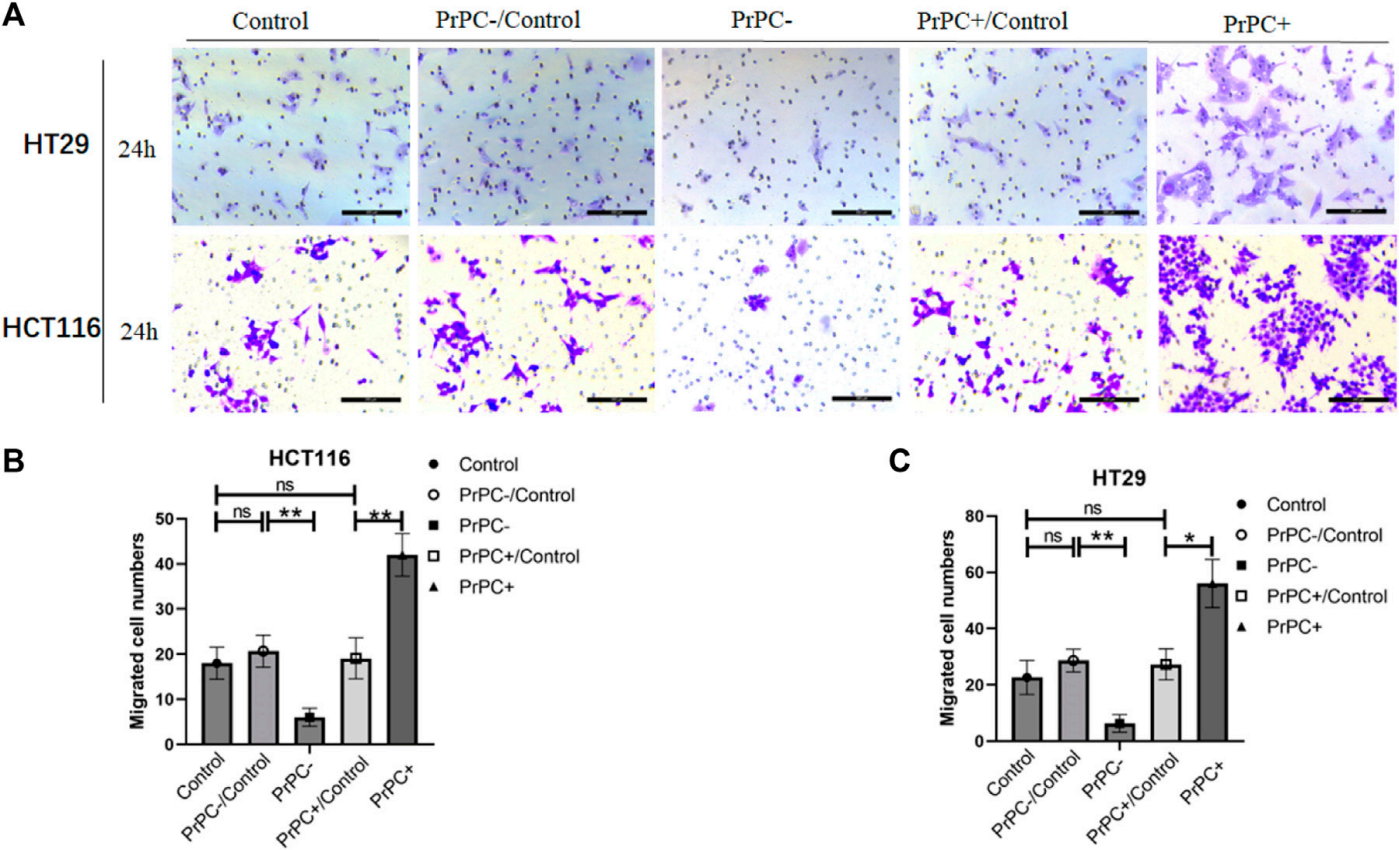
The invasion of PrPC on CRC cell lines and the control group were detected by the cell invasion assay. ns, no significance. *p < 0.05, **p < 0.01, ***p < 0.001 for comparison with the control group in **(A–C)**.

*In-vitro* cell invasion assays were conducted to explore the influence of varying PrPC expression levels on CRC invasion (Figure 10A). As presented in Figure 10B, the results demonstrated a decrease that is significant in the invasion ability of HCT116 cells in the PrPC- group compared to that in the PrPC- control group, and the invasion ability decreased by 6% (*p* < 0.01). In contrast, the invasion ability of the PrPC+ group was remarkedly increased compared to that of the PrPC+ control group, and the invasion ability increased by 42% (*p* < 0.01). Similar patterns were observed in HT29 cells, the invasion ability decreased by 6.33% in PrPC- group compared to control group (*p* < 0.01), and the migration ability increased by 56% in PrPC+ group compared to control group (*p* < 0.05) (Figure 10C).

### 3.8 Effects of different PrPC expression levels on EMT-related protein expression in CRC cell lines

**FIGURE 11:**
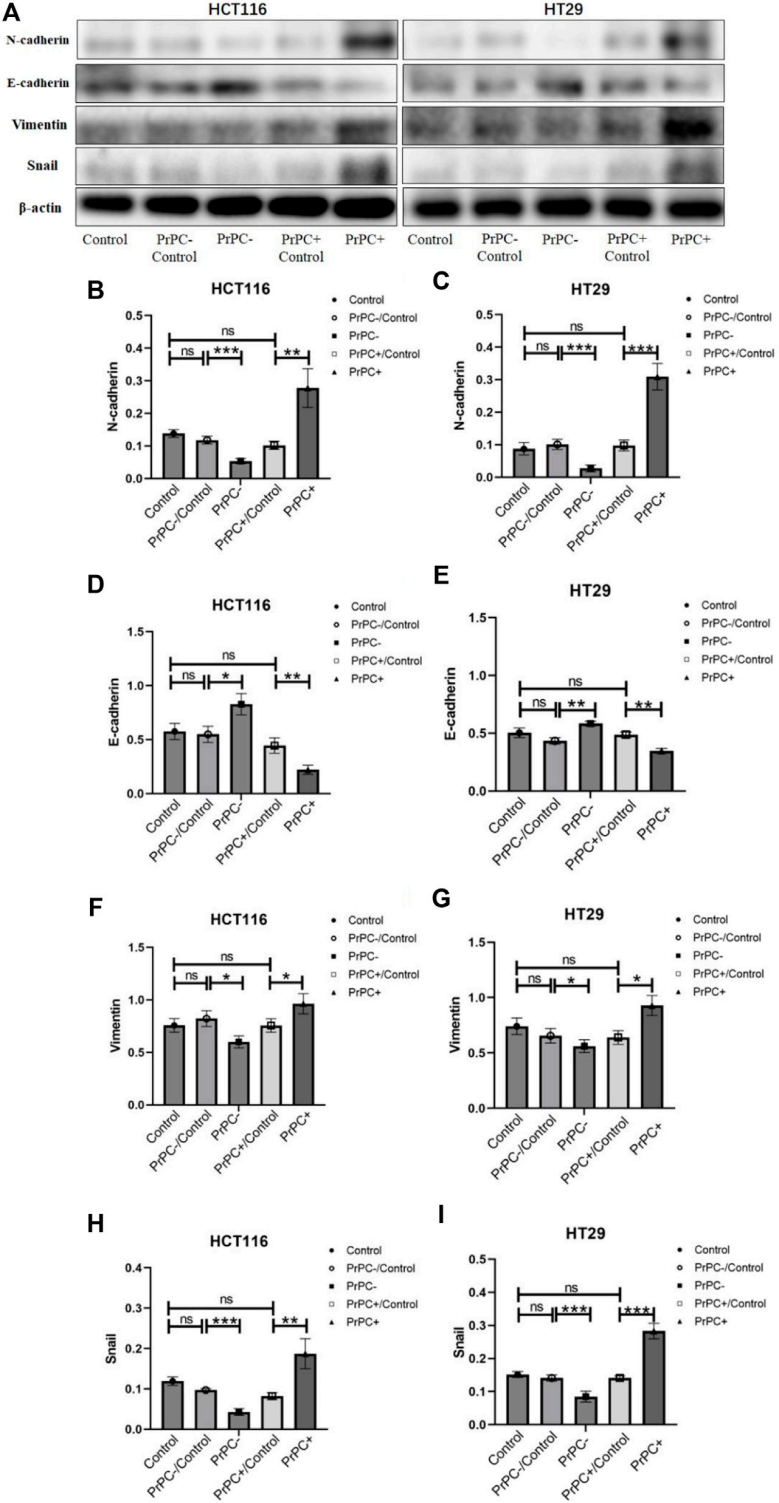
N-cadherin, E-cadherin, Vimentin, and Snail were detected by western blot in different PrPC expression levels of CRC cell lines and the control group. ns, no significance. *p < 0.05, **p < 0.01, ***p < 0.001 for comparison with the control group in **(A–I)**.

In Figure 11A, the expression of EMT-related proteins (N-cadherin, E-cadherin, Vimentin and Snail) in CRC cell lines with varying PrPC expression levels was detected using western blot. The results (Figures 11B–I) indicated that silencing the PrPC gene led to a declined expression of N-cadherin (both *p* < 0.001), Vimentin (both *p* < 0.05) and Snail (both *p* < 0.001) and a surge in the expression level of E-cadherin (*p* < 0.05, *p* < 0.01) in HCT116 and HT29 cells. Conversely, when the PrPC gene was overexpressed, the expression of N-cadherin (*p* < 0.01, *p* < 0.001), Vimentin (both *p* < 0.05) and Snail (*p* < 0.01, *p* < 0.001) increased, and E-cadherin decreased (both *p* < 0.01) in both CRC cell lines. No significant difference was observed in the expression of N-cadherin, E-cadherin, Vimentin and Snail between the blank group and the control group.

## 4 Discussion

The PrPC, encoded by the gene PRNP, is responsible for producing the major prion protein and exhibits widespread expression in various tissues, with specifically high levels found in the CNS (Tang et al., 2016; Go and Lee, 2020). Recently, remarkable studies have affirmed a high expression of PRNP(PrPC) in different types of cancers (Gil et al., 2016; Luo et al., 2017; Atkinson et al., 2019; Lin et al., 2020), consistent with our findings. Our investigation of PRNP(PrPC) expression in pan-cancer revealed significant upregulation in various cancer types, such as BRCA, COADREAD, ESCA, and STAD. The role and function of PRNP(PrPC) in influencing the proliferation, apoptosis, invasion, and metastasis of different cancer types indicate its potential as a promising therapeutic target involved in the therapeutic management of cancer.

Furthermore, we conducted an analysis to determine correlations between PRNP(PrPC) expression and TME, immune cell infiltration, and immune checkpoints. The TME comprises multiple immune cell types that function in either clearing tumor cells or promoting tumor immune escape, significantly impacting tumor prognosis (Bindea et al., 2013; Huntington et al., 2020). We found a positive link between PRNP(PrPC) expression and several cell types, particularly macrophages, neutrophils, CAFs, and CD8^+^ T cells while observing a negative correlation with plasma cells and regulatory T cells. With the rapid development of immune checkpoint inhibitors-based immunotherapies, there is an increasing need to develop biomarkers capable of predicting patient responsiveness to these treatments (Ribas and Wolchok, 2018). CTLA-4 and PD-1 have been identified as crucial regulators of T-cell reactions and exhibit promising potential as therapeutic targets for cancer treatment (Anderson et al., 2016; Korman et al., 2022). Recently, Giacomelli et al. (2023) found that immune suppressive microenvironment of mismatch repair proficient (pMMR) CRC characterized by dense infiltration of TAMs, occurrence of TANs, T-cell exhaustion, and interferon-γ unresponsiveness by host and tumor cells. Notably, we observed a significant association between PRNP(PrPC) and the majority of immune checkpoint molecules across various cancer types, particularly robust correlations with CD274 and C10orf54.

The expression of PRNP(PrPC) was observed to vary in both CRC tissues and their corresponding normal colorectal tissues, with a significantly higher positive expression rate noted in CRC compared to normal colorectal tissues. Moreover, we identified an association between the level of PRNP(PrPC) expression in CRC tissues and various clinicopathological features such as TNM stage, tumor invasion depth, tumor differentiation degree, presence of vascular invasion, and lymph node metastasis. Importantly, our findings also affirm a correlation between high PRNP(PrPC) expression levels and poor prognosis among CRC patients (Du et al., 2022). Subsequent research has revealed that the downregulation of PRNP(PrPC) expression can increase the sensitivity of HT29 cells to the chemotherapy drug cisplatin and promote cisplatin-induced apoptosis. The mechanism may be linked to the upregulation of apoptotic proteins Bax as well as caspase-3, along with the downregulation of the anti-apoptotic protein Bcl-2 (Du et al., 2019). Considering that sustained proliferative ability is considered one of the fundamental characteristics of cancer cells, extensive research has been conducted on the role of PrPC in promoting cancer cell proliferation. Early evidence supporting the involvement of PrPC in driving cancer cell growth was provided by Daiming Fan’s team through their investigation using gastric cancer cell lines SGC7901 and AGS (Liang et al., 2007). Chieng and Say (2015) demonstrated that overexpression of PrPC in LS 174T cells promotes cell growth and proliferation, while the proliferation of colon cancer cells significantly reduced after siRNA knockdown of PrPC in DLD-1 and SW480 CRC cell lines. Yun et al. (2016) found that HT29 cells treated with fucoidan exhibited decreased cell proliferation, diminished levels of the anti-apoptotic protein Bcl-2, and heightened levels of pro-apoptotic proteins Bax, cleaved caspase-3, and cleaved PARP1. Furthermore, the fucoidan-induced alterations in cell proliferation, apoptosis, and migration were further enhanced by downregulating PrPC expression using si-PRNP. Administering si-PRNP along with fucoidan via intraperitoneal injection resulted in reduced tumor volume and proliferation in Balb/c nude mice. These findings imply that the improved antitumor efficacy detected may be attributed to a decrease in angiogenesis. Similarly, we observed that the overexpression of PrPC in HCT116 and HT29 cell lines can promote the proliferation of CRC cells, and with prolonged cell culture time, the proliferation capacity of both CRC cells increased. This suggests a close relationship between PrPC expression levels and the proliferation of CRC cell lines.

Metastasis, the primary cause of death in cancer patients, signifies an advanced stage of malignancy. This progression encompasses various mechanisms, including the movement and infiltration of malignant cells, which are characteristic traits (Tahtamouni et al., 2019). EMT, a crucial factor in tumor invasion and metastasis as well as embryonic development, serves as a primary molecular mechanism facilitating the enhancement of metastasis and invasion during cancer promotion (Hodge et al., 2018). The orchestration of the EMT program involves key transcription factors consisting of Slug, Snail, Twist, as well as ZEB1 or ZEB2 (Lu and Kang, 2019). Du et al. (2013) found that mesenchymal genes (Twist and N-cadherin) were significantly upregulated, while the epithelial marker E-cadherin was downregulated in PrPC overexpressing colorectal cancer stem cells (CCSCs), whereas knockdown of PrPC resulted in a reversed expression pattern. In addition, their results further confirmed the correlation of PrPC with the expression of EMT-related molecules by using double immunofluorescence staining and western blot analysis. Through further research, the authors concluded that PrPC regulated the EMT phenotype by modulating Twist, and it was positively correlated with the mesenchymal properties of cells from CRC patients. Moreover, they found that PrPC promoted EMT via the ERK2 (MAPK1) signaling pathway and conferring high metastatic capacity. In our study, Wound healing assay and Transwell assay were employed to explore the impact of PrPC expression on the migration ability of CRC cell lines. It was found that high PrPC expression can promote increased migration ability of HCT116 and HT29 cell lines and may be related to EMT-related proteins (N-cadherin, E-cadherin, Vimentin and Snail). Notably, an increase in PrPC expression led to elevated N-cadherin, Vimentin and Snail expression and decreased E-cadherin expression. Conversely, knockdown of PrPC reversed EMT and reduced cell proliferation, migration, and invasion, while down-regulating N-cadherin, Vimentin and Snail and up-regulating E-cadherin. The EMT and TGF-β axes are the two main pathways to distinguish the C4 and CMS4 subgroups in CRC, and they also found that EMT and TGF-β signaling pathway feature among those that are correlated with PRNP gene expression (Le Corre et al., 2019). De Lacerda et al. observed that the migration as well as the invasion of CRC cell lines were stimulated by HOP in a PrPC-dependent manner, and the impact of HOP on cell migration and invasion is mediated through the phosphorylation of the ERK1/2 pathway (de Lacerda et al., 2016). These results propose that the ERK1/2 pathway participates in the HOP-driven invasion of CRC cells. In a study on PrPC’s role in EMT formation, Wang Q. et al. (2012) found that reducing the transcription of PrPC could down-regulate the expression of special AT-rich sequence-binding protein-1 (SATB1) through the Fyn-SP1-SATB1 pathway, thereby reducing the metastatic ability of CRC cells and decreasing their distant metastasis *in vivo*. Based on these findings, it is observed that PrPC may function in the process of distant metastasis of CRC, providing a potential new therapeutic target for patients with distant metastasis of CRC.

## 5 Conclusion

In summary, the results of our study inferred that PRNP(PrPC) is an immune-related prognostic biomarker and has the potential to serve as a prognostic indicator for CRC immunotherapy, while also promoting proliferation, migration, and invasion. Additionally, the overexpression of PRNP(PrPC) can promote the occurrence of EMT in CRC cells. The mechanism may be linked to the upregulation of N-cadherin, Vimentin and Snail and downregulation of E-cadherin. However, its specific signaling pathway is still unclear and warrants further investigation.

## Data Availability

The datasets presented in this study can be found in online repositories. The names of the repository/repositories and accession number(s) can be found in the article/Supplementary Material.
