# Supplementary material for: PRNP is a pan-cancer prognostic and immunity-related to EMT in colorectal cancer
Source: Front Cell Dev Biol. 2024 Aug 5;12:1391873. doi: 10.3389/fcell.2024.1391873 (PMC11336278; doi:10.3389/fcell.2024.1391873)
Supplement: Supplementary file 1 [file Table1.DOCX]

Supplementary Table 1. The sequences of shRNAs

| shRNAs | RNA Interference Target Sequence |
| --- | --- |
| *PRNP* | 5'-CACCGCAGTTGTGAAAGCACCATCACGAATGATGGTGCTTTCACAACTGC-3'  5'-AAAAGCAGTTGTGAAAGCACCATCATTCGTGATGGTGCTTTCACAACTGC-3' |
